# Mechanistic Understanding of Cell Recognition and Immune Reaction via CR1/CR3 by HAP- and SiO_2_-NPs

**DOI:** 10.1155/2020/7474807

**Published:** 2020-04-21

**Authors:** Tingting Ding, Jiao Sun

**Affiliations:** Shanghai Biomaterials Research & Testing Center, Ninth People's Hospital, Shanghai Jiaotong University School of Medicine, No. 427, Ju-men Road, Shanghai 200023, China

## Abstract

Nanodrug carrier will eventually enter the blood when intravenously injected or in other ways. Meanwhile, a series of toxic effects were caused to the body with the formation of nanoparticle protein corona. In our studies, we try to reveal the recognition mechanism of nanoparticle protein corona by monocyte and the damage effect on immune cells by activated complement of hydroxyapatite nanoparticles (HAP-NPs) and silicon dioxide nanoparticles (SiO_2_-NPs). So expressions of TLR4/CR1/CR were analyzed by flow cytometry (FCM) in order to illuminate the recognition mechanism of nanoparticle protein corona by monocyte. And the expression of ROS, cytokines, adhesion molecules, and arachidonic acid was measured when THP-1 and HUVECs were stimulated by NP-activated complement. The results showed that HAP-NPs can be recognized by the opsonin receptor (iC3b/CR3) model, while plasma protein, opsonin receptor, and Toll-like receptors are all likely launch cell recognition of SiO_2_-NPs. And it was considerate that NP-activated complement can damage THP-1 and HUVECs, including oxidative stress, inflammation, and increased vascular permeability. So the surface of nanodrug carrier can be modified to avoid being clear and reduce the efficacy according to the three receptors (TLR4/CR1/CR3).

## 1. Introduction

Nanoparticles (NPs) can finally enter the blood through respiratory, digestive tract, and skin permeation or injection and exist in the form of protein corona. In early effective stage, nanoparticles preferentially absorb immune globulin IgG and complement protein [1]. Once the size and surface composition of nanoparticles were changed, there will be side effects on absorption and transfer of immune toxicity. For example, as a complement protein of high concentration in plasma, nanoparticles can accelerate the uptake by macrophage when absorbing opsonin. Through opsonization, nanoparticles were rapidly eliminated via the reticuloendothelial system [2]. It had been confirmed that nanomaterials absorbing IgG can induce uptake through Fc receptor of macrophage and thus causing elimination of NPs in the blood [3]. Nanoparticle uptake of macrophage was developed through absorbed complement and receptor of macrophage, including CR1 and CR3 [4, 5]. Therefore, with the formation of nanoparticle protein corona, NPs may be gathered in the liver or spleen, and meanwhile, a series of toxic negative effects were caused to the body.

In the researches of macrophage identifying nanoparticle protein corona, Karmali and others figured out that there was a direct identifying mode which was through TLRs mediation besides opsonin-dependent mode induced by CR1 or CR3. Nanodrug carrier identification and devourment of monocyte and macrophage were finally promoted, thus resulting in contagion effects of systemic bacterial or foreign matter [6–8]. Then, once the drug carrier nanoparticles enter the blood, the body chose to identify and eliminate nanoparticles or extend the time of drug carrier in the blood to enhance efficacy. Any drug carrier nanoparticle would suffer from this choice and risk during application, and there were existing researches verifying elimination risks by macrophage for nanoparticles such as lipidosome, polylactide, and PMMA [9]. Therefore, to avoid identification of nanodrug carrier by monocyte for maximal efficacy, it is very necessary to explore mechanisms among nanoparticles, complement system, and monocyte.

Meanwhile, complement was an important part of the immune system. Once nanoparticle corona was identified and phagocytosed by monocyte or macrophage via activated complements, cascade amplified inflammation and immunological effects would be bound to be caused. C3a, C4a, C5a, and other complement active molecules would play nonspecific immunological effects via monocyte in blood. C5a was the strongest chemotactic factor which can aggregate and activate leukocytes to injured parts, producing leukotrienes B4 (LTB4), platelet-activating factor (PAF), and other inflammatory factors [10–12]. LTB4 and PAF together aggravated the assembly, activation, and injury of neutrophils and carried positive feedback regulation to immune response [13, 14]. At the same time, the activated neutrophils would induce highly expressed adhesion molecules of vascular endothelial cells, thus leading to injury and apoptosis of endothelial cells. Besides aggravating nonspecific immune response, endothelial cell injury was also a signal for thrombosis induction [15, 16]. There were literature verifying the risks of thrombosis after nanoparticle contacting with blood through fibrous protein (Fg), platelet count and aggregation, blood clotting time and ATP release, and other aspects. In preliminary studies, we have verified that hydroxyapatite nanoparticles (HAP-NPs) and silicon dioxide nanoparticles (SiO_2_-NPs) could activate complement through different pathways and whether coagulation can be initiated because of injured endothelial or immune cells by the activated complements finally. Therefore, it is important to study the relationship for nanoparticles, activated complements, and immune cells in order for design, surface modification, or application of drug carrier nanoparticles.

Both HAP-NPs and SiO_2_-NPs are inorganic nanoparticles and attracting great attention for drug delivery because they are advantageous in stable structure, easy preparation, and good biological compatibility of the material self. As researched objects, identification mechanism will be explored from the angle of interaction among nanoparticles, complement, and immune cells caused by HAP-NPs and SiO_2_-NPs. Finally, it is anticipated that pesticide effect could be increased through reducing identification and elimination by the immune system via different surface modification of NPs.

## 2. Materials and Methods

### 2.1. Human Serum Collection and Characterization of NPs

**Figure 1:**
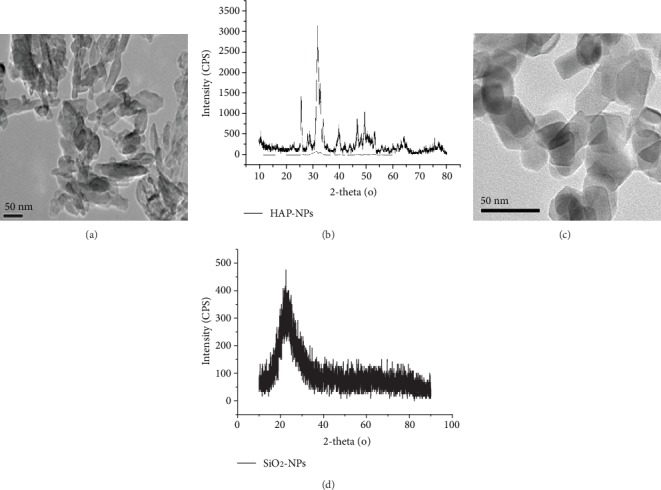
Characterization of HAP-NPs and SiO_2_-NPs. (a) TEM image showing size distribution for HAP-NPs; (b) XRD patterns for HAP-NPs. (c) TEM image showing size distribution for SiO_2_-NPs; (d) XRD patterns for SiO_2_-NPs.

To harvest serum, blood which was donated by healthy volunteers was allowed to clot for 60 min at RT before centrifugation at 1000 rcf for 15 min at RT. Serum was freshly collected for each experiment. And the average size of HAP-NPs (Nanjing Emperor Nano Material Company Ltd, China) and SiO_2_-NPs (Sigma Chemical Company St. Louis, MO, USA) was confirmed by transmission electron microscopy (TEM, JEM-2010, JEOL Ltd, Tokyo, Japan). The structure of HAP-NPs and SiO_2_-NPs was confirmed by X-ray diffraction (XRD, Rigaku Corporation, Japan). The results of XRD showed that the two nanoparticles were HAP-NPs and SiO_2_-NPs, respectively. And rod-like HAP-NPs (short diameter: 20 nm/long diameter: 80 nm) and spherical SiO_2_-NPs (20 nm) were well distributed (Figure 1).

### 2.2. Culture of Monocyte and Endothelial Cells

THP-1 cells were grown in suspended state in high glucose DMEM medium with 10% fetal bovine serum and cell density was kept at 2 × 10^5^‐10^6^ cell per mL. Fresh umbilical cord (provided by the Obstetrics Department in the International Peace Maternity and Child Health Hospital of China Welfare Institute) of healthy infants who received cesarean section and were without hepatitis and HIV contagion in aseptic conditions was taken. Use PBS to irrigate veins and inject 0.1% type I collagenase (US Sigma company) to umbilical veins for digestion. Irrigate and collect human umbilical vein endothelial cells (HUVECs) and resuspend with ECM medium with 10% fetal bovine serum to adjust to appropriate concentration for culture. Once the primary cells were fused over 80%, passage can be initiated and generally 3^rd^ to 6^th^ generation cells are taken for experiment.

### 2.3. NPs Activating Complement System

HAP-NPs (10 *μ*g/mL) and SiO_2_-NPs (6 *μ*g/mL) were suspended in Ca^2+^/Mg^2+^-free phosphate-buffered saline (PBS). Then, 10 *μ*L of each of veronal buffer (VB), human plasma, and NPs was combined as a test sample. Reaction components in the negative control (NC) group were PBS without NPs, human serum, and VB. And EDTA (0.15 mM)/EGTA (1 mM) was replaced by PBS for the inhibitor test of monocyte effects. After incubation at 37°C for one hour, centrifuge the cells at high speed 12000 rpm for 20 min and then take the supernatant. Wash EDTA for precipitation use for three times, and then, resuspend and collect particles absorbing plasma proteins.

### 2.4. Cellular Recognition Mechanism Induced by NPs Activating Complement

Inoculate THP-1 to plates with six holes at 5 × 10^5^ per hole depth, and there were three parallel samples in each group and culture for 24 h. Dilute complement supernatant in negative control (NC) and two particles (HAP/SiO_2_-C group) with high glucose DMEM medium as 1 : 10 ratio. Meanwhile, dilute particles absorbing plasma proteins with high glucose DMEM as 0.1 *μ*g/mL final concentration (HAP/SiO_2_-NPs-Pro group). Expose samples in each experimental group to THP-1 cells and incubate at 37°C for 24 h. Collect and wash the resuspension and add flow antibody TLR4 (CD284-APC)/CR1 (CD35-PE)/CR3 (CD11b-APC) (Miltenyi Biotec Company, Germany) and incubate at 4°C for 10 min avoiding light. Centrifuge with 300 g for 10 min, then discard the supernatant and wash with buffer solution for two times. Finally, after 200 *μ*L buffer for resuspension, use a flow cytometer to analyze the expression of each membrane protein of monocyte.

### 2.5. Effects on Monocyte through Activating Complement of NPs

Inoculate THP-1 to plates with six holes at 5 × 10^5^ per hole depth, and there were three parallel samples in each group and culture for 24 h. Centrifuge cells from each hole and discard supernatant, then add medium from each group with 10% complement. Continue to culture for 24 h and collect, wash, and resuspend. Add 1 mL DCFH-DA application solution (2.5 *μ*M) (Beijing Applygen Genetic Technology Co., Ltd) which was diluted in medium as 1 : 10000 to each hole. Stain at 37°C for 30 min avoiding light. Wash, centrifuge, and collect cells to examine reactive oxygen expression of monocyte with flow cytometer. The results were indicated with mean fluorescence intensity (MFI).

Collect cells from each group using the above methods and use the extraction kit for cytoplasm and nuclear protein (purchased from US Pierce company) to extract cytoplasm and nuclear protein. Apply Western blot to check the changes of monocyte NF-*κ*B (purchased from US Santa Cruz company) expression. In addition, after 24 h of monocyte induction by nanoparticles activating complement, centrifuge and collect supernatant and examine expression of monocyte TNF-*α*/IL-6 (Shanghai ExCell Biology Inc.), histamine (purchased from US IBL company), and PEG/LTB (US Abcam company) with ELISA.

### 2.6. Effects on HUVECs through Activating Complement of NPs

Take HUVECs in logarithmic growth phase and adjust cell density to 5 × 10^5^/mL and inoculate to plate with six holes. Culture at 37°C for 24 h and add medium with 10% complement from each group for a 24 h continual culture. Collect cells from each hole and collect and wash for resuspension. Add flow antibody E-selectin (CD62E APC)/ICAM-1 (CD54 PE)/VCAM-1 (CD106 FITC) (Miltenyi Biotec Company, Germany) after 10 min incubation at 4°C avoiding light, centrifuge and wash, and then analyze expression of each adhesive molecules with flow cytometer. Meanwhile, collect supernatant in each hole and examine expression of MCP-1 and IL-8 (Shanghai ExCell Biology Inc.) with the ELISA kit.

## 3. Results and Discussions

### 3.1. Cellular Recognition Mechanism Induced by NPs Activating Complement

In anti-infection immune reaction, TLR4 (CD284), CR3 (CD11b, iC3bR), and CR1 (CD35, C3b/C4bR) all act significant biological effects. TLR4 (CD284) was a LPS receptor of transmembrane and could transcribe multiple cytokines and adhesive molecules through the NF-*κ*B signal system, leading to the cascade amplification of inflammatory reaction [17]. As one of the most significant devour receptors as professional macrophage, CR3 (CD11b, iC3bR) was of strong recognition capability of broad spectrum ligand. It can promote adhesion, transfer, and devourment of macrophage, while CR1 (CD35, C3b/C4bR) could specifically eliminate immune complex (IC) combined with C3b/C4b [18].

To explore the identified function of nanoprotein corona, three experimental groups were designed in this research: (1) HAP/SiO_2_-NPs-Pro group: nanoparticles be deposited of activated complement proteins; (2) HAP/SiO_2_-NPs group: nanoparticles which were not incubated with human serum and were without adhesive protein on the surface; (3) HAP/SiO_2_-C group: activated complement supernatant after incubation and centrifuge of nanoparticles and human serum. On the basis of the above experimental group, we explored a possible mechanism identifying nanoparticle protein corona of monocyte through studying the changes of cell surface TLR4 (CD284), CR1 (CD35), and CR3 (CD11b), to see whether its simple nanoparticles, simple plasma proteins, and coordination of nanoparticles and plasma proteins were required.

#### 3.1.1. Cellular Recognition Mechanism Induced by HAP-NPs

**Figure 2:**
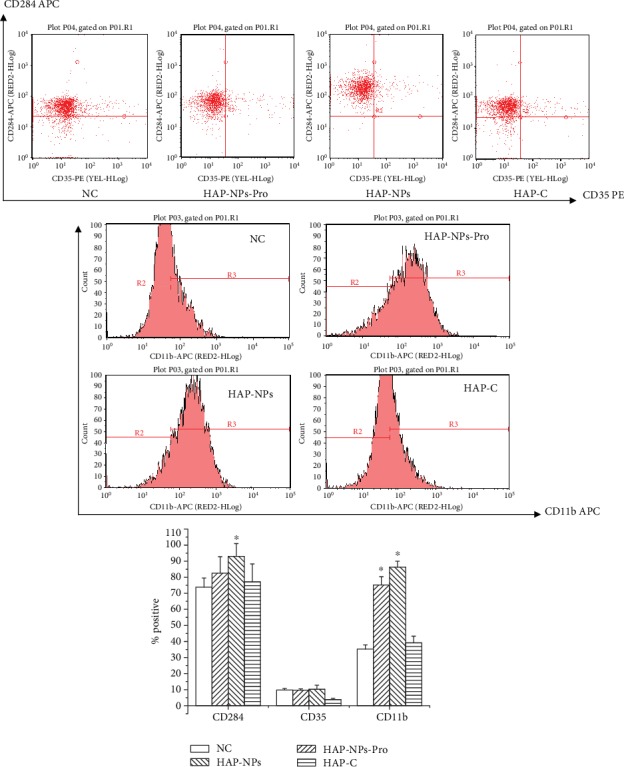
Scheme of possible mechanisms of HAP-NPs recognition by THP-1. (1) HAP-NPs-Pro: nanoparticles be deposited of activated complement proteins; (2) HAP-NPs: nanoparticles which were not incubated with human serum and were without adhesive protein on the surface; (3) HAP-C: activated complement supernatant after incubation and centrifuge of nanoparticles and human serum (^∗^*P* < 0.05 versus NC group).

According to experimental results (Figure 2), we discovered that there were no obvious changes of CR1 (CD35) in each experimental group of HAP-NPs. This proved that there was no immune complex (IC) combined with C3b/C4b in HAP-NPs and activated complement. Thus, HAP-NPs was not identified and devoured through this signal pathway.

According to the results of TLR4, significant expression of TLR4 (CD284) was caused by HAP-NPs without protein encapsulation. Positive expression percentage was 93.04 ± 7.89, and there was obvious difference comparison with the negative control group (*P* < 0.05). And TLR4 (CD284) in the HAP-NPs-Pro group was not greatly expressed (*P* > 0.05), which was equivalent to the activated complement supernatant group without particle (HAP-C). Our results demonstrated that there was TLR4 ligand on HAP-NPs surface. And TLR4/NF-*κ*B signal pathway can be solely initiated and inflammatory reaction of monocyte was caused, which was consistent with literature reports on monocyte injury caused by HAP-NPs [19]. When HAP-NPs were incubated with human serum, the absorbed plasma proteins (activated complements) occupied the location of TLR4 ligand. According to the results, specific surface modification can be designed like TLR4 ligand in order to escape phagocytose in the immune system for NPs in the application of drug delivery [20, 21].

Meanwhile, HAP-NPs-Pro could lead to obvious expression of CR3 (CD11b) and its positive expression percentage was 75.18 ± 5.17. There was remarkable difference when compared with negative control (*P* < 0.05). These results showed that HAP-NPs initiated signal pathway of iC3b/CR3, then HAP-NPs were devoured and eliminated via absorbed iC3b. But HAP-NPs without protein encapsulation could also lead to obvious expression of CR3 (CD11b) and its positive expression percentage was 86.34 ± 3.64, which was remarkably different when compared with negative control (*P* < 0.05). We deduced that CR3 can recognize ligands in broad spectrum because CR3 could also be bound to nonprotein products. Therefore, according to our results, HAP-NPs mainly were directly devoured and eliminated via the iC3b/CR3 model because of opsonic when entered into blood as drug carrier. TLR4/NF-*κ*B signal pathway initiating inflammation reaction will not be induced by HAP-NPs. So in the application of HAP-NPs in drug delivery, surface modification should be considered to reduce the absorption of iC3b. And iC3b/CR3 model may avoid initiation for eliminating NPs finally [22, 23].

#### 3.1.2. Cellular Recognition Mechanism Induced by SiO_2_-NPs

**Figure 3:**
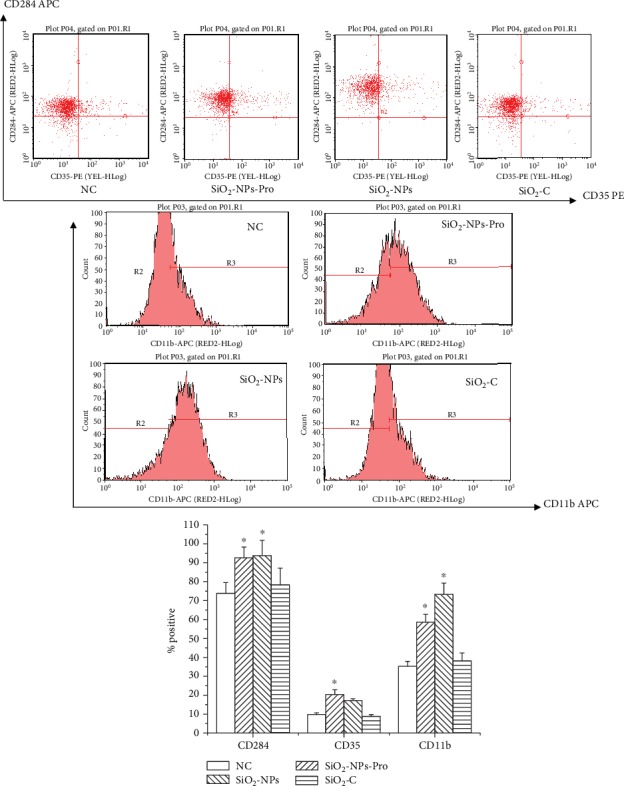
Scheme of possible mechanisms of SiO_2_-NPs recognition by THP-1. (1) SiO_2_-NPs-Pro: nanoparticles be deposited of activated complement proteins; (2) SiO_2_-NPs: nanoparticles which were not incubated with human serum and were without adhesive protein on the surface; (3) SiO_2_-C: activated complement supernatant after incubation and centrifuge of nanoparticles and human serum (^∗^*P* < 0.05 versus NC group).

Active fragments (SiO_2_-C) after complement activation induced by SiO_2_-NPs posed no obvious effects on expression of three receptors on the surface of THP-1 cells (Figure 3). Positive cellular expression percentage of monocyte surface TLR4 (CD284) in the SiO_2_-NPs-Pro and SiO_2_-NPs experimental groups, respectively, reached 92.62 ± 5.69 and 93.74 ± 8.12, which were greatly elevated when compared with the negative group (*P* < 0.05). Meanwhile, SiO_2_-NPs-Pro and SiO_2_-NPs could all lead to remarkable expression of CR3 (CD11b) (*P* < 0.05). Differing from HAP-NPs, only monocyte surface CR1 (CD35) in the SiO_2_-NPs-Pro group was greatly elevated and positive cellular percentage was 20.32 ± 2.54.

Therefore, differing from HAP-NPs, SiO_2-_NPs-Pro could cause high expression of different receptors TLR4, CR3, and CR1 at the same time. Hence, SiO_2_-NPs were faced with risks of being identified by multiple mechanisms, including opsonin by CR3, the NF-*κ*B signal system via TLR, or immune complex by CR1 once drug carrier entered the blood. So the interaction among different proteins should be considered synthetically when modification in surface in order for escaping [24, 25]. Meanwhile, two nanoparticles without protein encapsulation could all lead to TLR4 expression and its mechanism was to cause inflammatory reaction through inducing TLR4/NF-*κ*B signal pathway. This further reminded the appraisal of immunological effects of HAP/SiO_2_-NPs through activating complement.

### 3.2. Effects on Monocyte through Activating Complement of NPs

#### 3.2.1. Damage Effect on Monocyte

C3a, C4a, C5a, and other complement active molecules could give play to nonspecific immune response through monocyte; therefore, complement activation might be one of the mechanisms for monocyte injury resulted in by nanoparticles [26]. To explore monocyte oxidative stress and inflammatory reaction caused by the complement system with C5a and other active molecules, we continued to culture THP-1 cells with complement serum after being activated by HAP and SiO_2_ nanoparticles.

**Figure 4:**
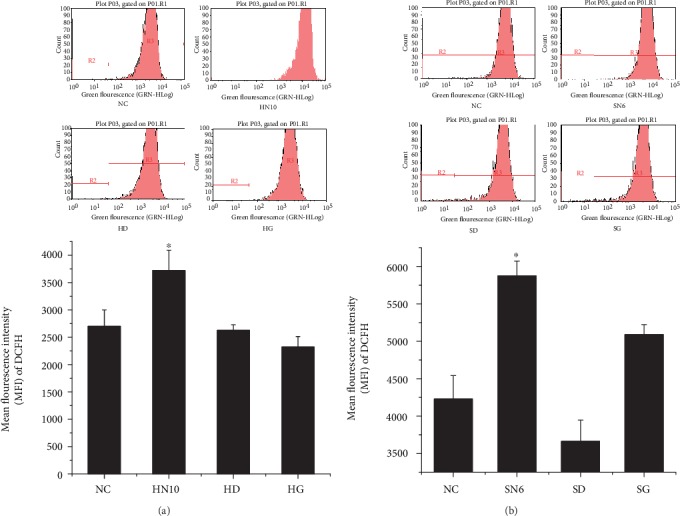
Effect of ROS in THP-1 by activated complement of NPs. (a) For HAP-NPS, HN10: HAP-NPs (10 *μ*g/mL); HD: HAP-NPs/EDTA (0.1 mM EDTA); HG: HAP-NPs/EGTA (1 mM EGTA). (b) For SiO_2_-NPs, SN6: SiO_2_-NPs (6 *μ*g/mL); SD: SiO_2_-NPs/EDTA (0.1 mM EDTA); SG: SiO_2_-NPs/EGTA (1 mM EGTA). ^∗^*P* < 0.05 versus NC group.

**Figure 5:**
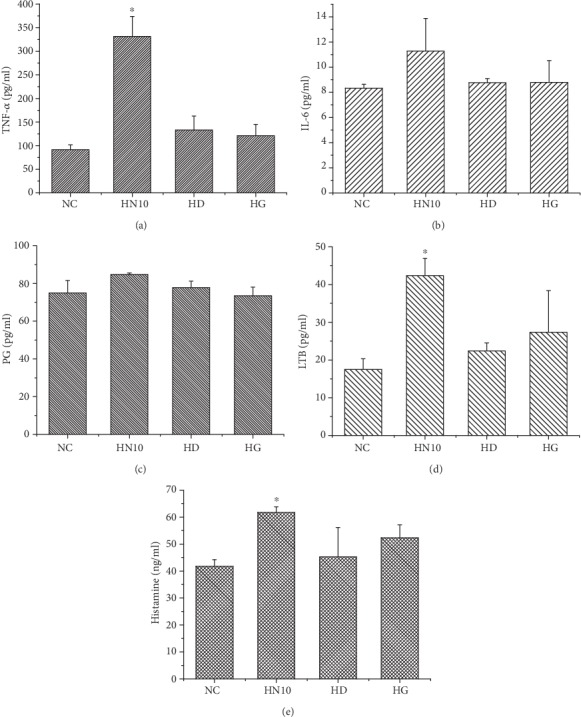
Effect of cytokines in THP-1 by activated complement of HAP-NPs. HN10: HAP-NPs (10 *μ*g/mL); HD: HAP-NPs/EDTA (0.1 mM EDTA); HG: HAP-NPs/EGTA (1 mM EGTA). ^∗^*P* < 0.05 versus NC group.

**Figure 6:**
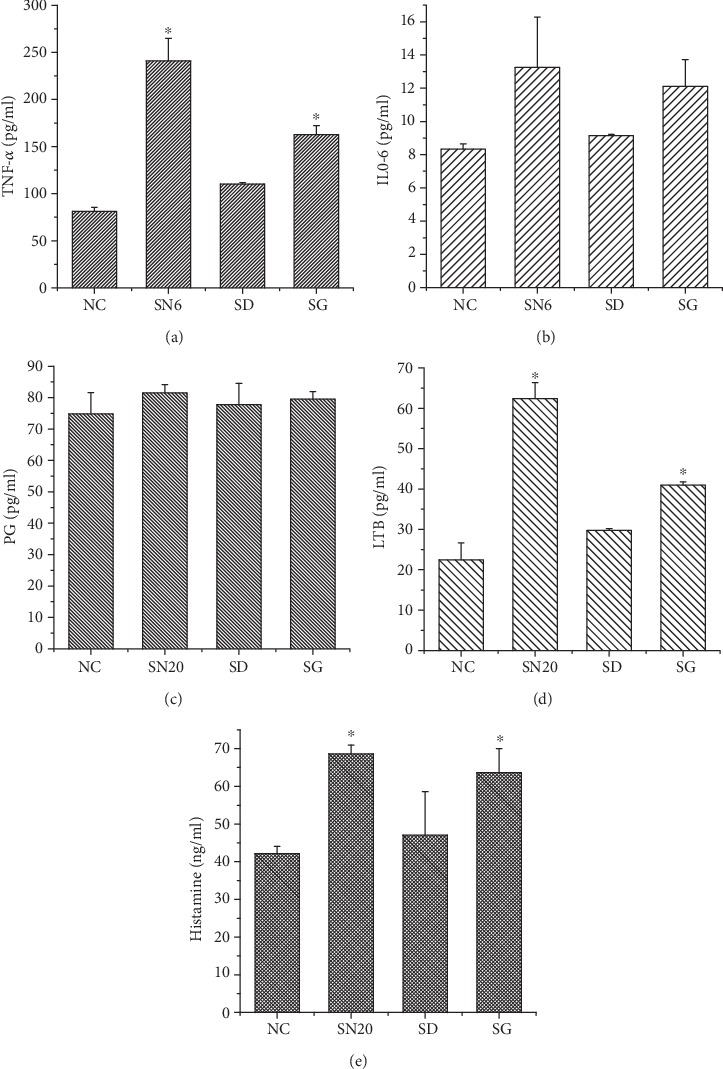
Effect of cytokines in THP-1 by activated complement of SiO_2_-NPs. SN6: SiO_2_-NPs (6 *μ*g/mL); SD: SiO_2_-NPs/EDTA (0.1 mM EDTA); SG: SiO_2_-NPs/EGTA (1 mM EGTA). ^∗^*P* < 0.05 versus NC group.

According to results (Figure 4), ROS MFI in HAP-NPs and SiO_2_-NPs were 3721.00 ± 372.64 and 5875.45 ± 195.68, respectively, which was significantly different compared with negative control (*P* < 0.05). At the same time, HAP-NPs and SiO_2_-NPs could all lead to obvious expression of TNF-*α* (*P* < 0.05) and concentrations were 331.66 ± 42.12 and 241.05 ± 23.70 (pg/mL), respectively (Figures 5 and 6). Overexpression of ROS indicated that the oxidation and antioxidation system of monocyte was imbalanced, causing oxidative stress reaction and thus leading to cell injury [27], while TNF-*α* was a significant cytokine in the inflammatory reaction and could further induce formation of IL-6, IL-8, and other cytokines that were participating jointly in acute reaction and fever reaction of the body. But compared with TNF-*α*, we have not discovered obvious expression of IL-6 (Figures 5 and 6). IL-6 can coordinate immune defense response once the body is injured. And EDTA/EGTA can reduce the above cytokines because of inhibition to complement activation. Therefore, our studies verified that HAP-NPs and SiO_2_-NPs could lead to inflammatory reaction through activating complement via monocyte, while monocyte did not initiate protection mechanism through IL-6 which will aggravate cascade effects [28–30].

**Figure 7:**
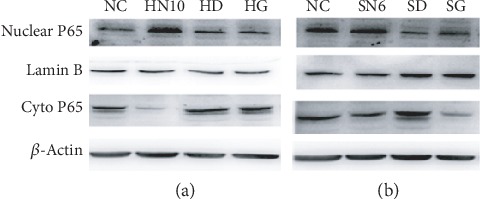
Effect of NF-*κ*B in THP-1 by activated complement of NPs. (a) For HAP-NPs, HN10: HAP-NPs (10 *μ*g/mL); HD: HAP-NPs/EDTA (0.1 mM EDTA); HG: HAP-NPs/EGTA (1 mM EGTA). (b) For SiO_2_-NPs, SN6: SiO_2_-NPs (6 *μ*g/mL); SD: SiO_2_-NPs/EDTA (0.1 mM EDTA); SG: SiO_2_-NPs/EGTA (1 mM EGTA).

At the same time, in the study of cellular recognition mechanism induced by activating complement, we figured out that two nanoparticles could induce TLR4/NF-*κ*B signal pathway to initiate inflammatory reaction of monocyte. Therefore, combining NF-*κ*B expression, this research further verified the mechanism of two nanoparticles HAP and SiO_2_ causing monocyte injury through complement activation. Experimental results showed that HAP and SiO_2_ could all induce NF-*κ*B transferring from the cytoplasm to the nuclei and being activated (Figure 7). NF-*κ*B was of important regulation effects to gene expression in immune and inflammation related reactions and it was an oxidative stress reaction related nuclear transcription factor. Once NF-*κ*B was activated from the cytoplasm to the nuclei, formation of target gene mRNA was induced and high ROS expression could further activate NF-*κ*B [31–33]. Therefore, complement activated by HAP-NPs and SiO_2_-NP could induce oxidative stress reaction of monocyte. And it is further verified that the NF-*κ*B pathway was one of the mechanisms causing monocyte injury by the nanoparticle-activated complement system.

#### 3.2.2. Chemotaxis Effect on Monocyte

In the activated complement system, C5a was the strongest chemotactic factor and under the effects of C5a activating phosphatidase and arachidonic acid (AA) could produce prostaglandin (PG), leukotrienes (LTB), and other products and induce agglomeration of more neutrophils, forming a positive feedback regulation [10]. LTB concentration in HAP-NPs and SiO_2_-NPs was 42.35 ± 4.58 and 62.38 ± 3.98 (pg/mL), respectively (Figures 5 and 6). There was obvious difference when compared with negative control (*P* < 0.05) while there was no significant difference regarding influence to PG (*P* > 0.05). In general, there were two pathways for activated metabolism of AA, including cyclooxygenase (CO) or lipoxygenase (LPO) pathway. Major metabolites of AA through CO pathway were PG and that through LPO was LTB [34]. Therefore, under activated complement effects of two nanoparticles, AA of THP-1 cells could produce inflammatory factor LTB through LPO, strengthening chemotactic effects. Therefore, complement active fragments activated by two nanoparticles were of obvious chemotactic effects to monocyte.

Meanwhile, C5a could enhance vascular permeability through further coordinating histamine release with LTB and aggravating agglomeration and adhesion of leukocytes. According to the ELISA results, HAP-NPs and SiO_2_-NPs could all cause obvious expression of histamine and concentration was 61.72 ± 2.08 and 68.63 ± 2.35 (ng/mL), respectively. Therefore, activated complement of two nanoparticles was one of the inducements further increasing vascular permeability. Meanwhile, it is indicated that effects of nanoparticles on endothelial cells were the inevitable key problems during the application of nanodrug carrier [13].

### 3.3. Effects on HUVECs through Activating Complement of NPs

Due to the continual exposure to products causing activated complement by nanoparticles, vascular endothelial cells played a key part in local inflammation reaction. They participated in nonspecific immune response through integrin, selectin, and other surface adhesive molecules. E-selectin was major molecules absorbed by mediated monocyte which was mainly expressed on the surface of endothelial cells. However, ICAM-1 and VCAM-1 were major family members of immune globulin on the surface of endothelial cells. Under normal conditions, endothelial cells rarely expressed E-selectin and VCAM-1 and only expressed ICAM-1 in very little amount. Once endothelial cells were activated, abundant expression of adhesion molecules would be caused and pose damages to the functions of endothelial cells [35]. Therefore, ICAM-1, VCAM-1, and E-selectin were main marks of endothelial cell activation and were closely correlated to inflammatory reaction.

**Figure 8:**
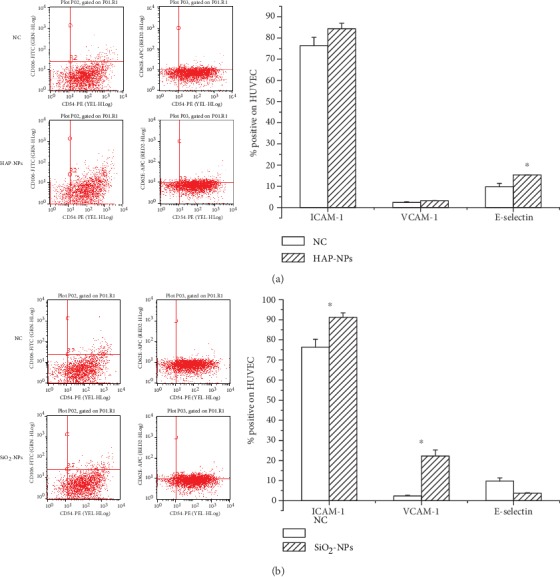
Effect of adhesion molecule in HUVECs by activated complement of NPs: (a) HAP-NPs and (b) SiO_2_-NPs. ^∗^*P* < 0.05 versus NC group.

In this research, when endothelial cells were induced 24 h later by the complement system of HAP-NPs and SiO_2_-NPs activation, only E-selectin (CD62E) in HAP-NPs group was positively expressed (15.29 ± 0.09). There was obvious elevation compared with negative control (9.78 ± 1.59) (*P* < 0.05) (Figure 8(a)). On the contrary, SiO_2_-NPs could lead to high expression of ICAM-1 (CD54) and VCAM-1 (CD106). And percentage was 91.20 ± 2.23 and 22.23 ± 3.05 (*P* < 0.05), respectively (Figure 8(b)). It is reported in literature that under the regulation of E-selectin and other selectins, leukocytes would repeatedly be absorbed to or detached from the surface of endothelial cells in rolling way. This temporary binding would activate the adhesive activity of leukocytes, thus leading to easier binding of ICAM-1, VCAM-1, and other adhesion molecules and creating conditions for tighter binding to endothelial cells and inducing them to transfer to endothelial cells to participate in physical and pathological course [16, 36]. Hence, when endothelial cells proliferated, the expression of ICAM-1 was the strongest. Referring to the results, endothelial cell injury caused by HAP-NPs activated complement was still in the early stage while SiO_2_-NPs activated complement has already caused proliferation of endothelial cells, enhancing cascade amplification effects of inflammatory reaction through highly expressed ICAM-1 and VCAM-1 [37].

**Figure 9:**
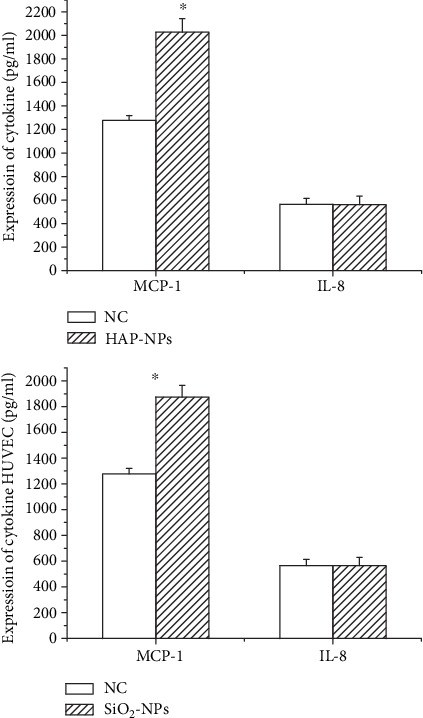
Effect of cytokines in HUVECs by activated complement of NPs (^∗^*P* < 0.05 versus NC group).

In the meantime, vascular endothelial cells would secrete cytokines with chemotactic effects such as IL-8, MCP-1, and MG-CSF, increasing the expression of integrin on surface of monocyte and enhancing metastasis speed. Under normal conditions, endothelial cells secreted fewer cytokines, but after injury, cytokines would be abnormally and highly expressed. Once endothelial cells were exposed to HAP-NPs and SiO_2_-NPs activated complement, the expression quantity of chemotactic factor MCP-1 after 24 h was 2027.65 ± 115.05 and 1874.09 ± 90.88 (pg/mL), respectively, showing obvious elevation compared with negative control (*P* < 0.05), but no changes were seen regarding L-8 expression (Figure 9). MP-1 belonged to the C-C/*β* subgroup and was mainly for monocyte chemotaxis. After monocyte activation, multiple interferon, interleukin, and other cytokines participated in the defense mechanism of the body. However, IL-8 belonged to the C-X-C/*α* subgroup and was mainly for neutrophil chemotaxis and neutrophils were mostly lysosome and related to devourment and digestive functions of the cells ^[130]^. Therefore, HAP-NPs and SiO_2_-NPs activated complement could further participate in local inflammatory reaction through monocyte chemotaxis by MCP-1 [27]. However, injury of vascular endothelial cells causes a series of problems such as coagulation disorder and thrombosis formation. That is why this result reminded us that we needed to pay attention to the potential risks of cardiovascular diseases caused by drug carrier nanoparticles.

## 4. Conclusion

**Figure 10:**
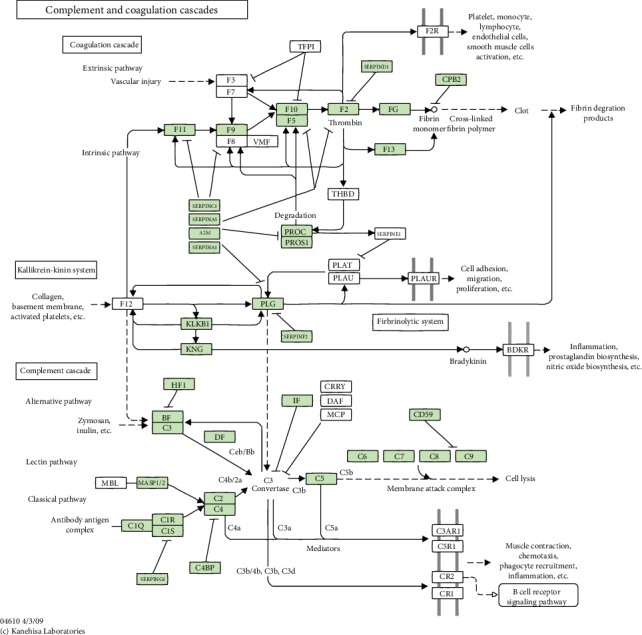
Map name of NPs: complement and coagulation cascades (Map ID: ko04610).

Starting from receptor-ligand angle, our studies found that HAP-NPs could be identified only through iC3b/CR3 (CD11b) pathway, while SiO_2_-NPs could be identified through the coordination of opsonic action and direct action. So different surface modification can be designed like TLR4 ligand or iC3b in order to escape phagocytose in the immune system for NPs in the application of drug delivery. Meanwhile, in preliminary studies of proteomics, annotation results of KEGG pathways (Map ID: ko04610, Figure 10) demonstrated that the location of absorbed complement proteins in signal pathway belonged to complement and coagulation cascades [38, 39]. Map of signal pathways showed that complement proteins were only a little part, and we saw that C3a/C4a/C5a would participate in a series of immunological effects such as leukocytes agglomeration, inflammatory reaction, and activation of B cells at the downstream. While on the upstream pathway, we also discovered that activated complements could activate platelets through receptor of platelet glycoprotein, thus initiating extrinsic coagulation pathways. Thus, it is indicated that when nanodrug carrier entered into the blood, complement activation was only a very small part. So it is an important breakthrough point that the damage of immune or coagulation cells can be researched through activation complements via nanodrug delivery.

## Data Availability

The data used to support the findings of this study are available from the corresponding author upon request. Data is expressed as the mean ± standard deviation (SD). Statistical analyses were performed with SPSS 12.0 software, and statistical comparisons were analyzed using the *t*-test. Differences were considered statistically significant when the p-value was less than 0.05.
